# Factors influencing the self-reported sense of deviation in adults with successful surgical outcomes for strabismus

**DOI:** 10.1186/s12886-019-1299-3

**Published:** 2020-01-06

**Authors:** Na Ji, Meiping Xu, Huanyun Yu, Jinling Xu, Xinping Yu

**Affiliations:** 1 The affiliated Eye Hospital of Suzhou Vocational Health College; i.e. the affiliated Suzhou Eye Hospital of Wenzhou Medical University, Suzhou, China; 2 The Eye Hospital of Wenzhou Medical University, No. 270, Xueyuan Xi Rd, Wenzhou, 325027 China

**Keywords:** Strabismus, Surgery, Self-sense, Outcome, Deviation

## Abstract

**Background:**

To determine whether a sense of deviation remains in adults with successful motor alignment who fulfil diplopia criteria after surgery and to examine the factors associated with this judgement.

**Methods:**

This was a retrospective study. Adult patients defined as having a successful outcome based on more than 1 year of post-operative follow-up visits were included in the study. The sense of deviation was determined at the last visit. Pre- and post-operative deviation and characteristics including age, gender, education level, occupation, diagnosis, size of deviation, extraocular movement (EOM), binocular function, and health-related quality of life (HRQOL) were recorded.

**Results:**

In total, 22 (24%) of the 91 adults with successful surgical outcomes reported a sense of deviation. No significant differences were noted between subjects with and without a sense of deviation regarding patient demographics, pre- and post-operative deviation, changes in deviation, sensory fusion or EOM. Subjects with a sense of deviation had an increased prevalence of and larger post-operative vertical deviation, poorer stereo function, and lower HRQOL scores than those with no sense of deviation. The presence of post-operative vertical deviation was associated with a sense of deviation.

**Conclusions:**

Approximately one-fourth (24%) of adults defined as having successful surgical outcomes who still had a sense of deviation exhibited worse stereo function, higher vertical deviation size and lower HRQOL scores. The presence of 3 to 5 prism dioptres(pd) of vertical deviation would be the main factor associated with a sense of deviation post-operatively.

## Background

Strabismus surgery in adults aims to improve ocular alignment, reduce diplopia, [1] and recover binocular function, [2, 3] with the benefit of improving psychosocial aspects of patients, e.g., social interactions, self-esteem, [4–7] and health-related quality of life (HRQOL) [8–10]. Patients with successful post-operative motor alignment have better HRQOL, which improves progressively after surgery [6, 7, 9–11]. The definition of successful alignment was inconsistent in previous studies(tropia within 8 to 12 prism dioptres (pd) horizontally and within 2 to 5 pd. or 10 pd. vertically) [12–16]. In addition, various discrepancies are consistently noted by patients and physicians in surgical outcome assessments [17]. Satterfield et al. found that greater than 80% of subjects thought that their eyes were still not perfectly straight; however, nearly all patients reported improved life after surgery [5]. Additionally, Hertle et al. reported highly subjective success in an adult group [16]. However, neither of these studies compared the patients’ subjective senses of their surgical outcome with their motor alignment [5, 16]. Self-esteem and characteristics associated with judgement in patients who undergo successful motor alignment after corrective surgery thus still require evaluation.

The present study evaluated the subjective self-sense of alignment with or without deviation in adults with successful motor alignment who fulfilled diplopia criteria after surgery, and the characteristic factors associated with their judgement were also studied.

## Methods

### Patients and methods

This retrospective study recruited adult patients who underwent corrective surgery at the Eye Hospital of Wenzhou Medical University between 1 January 2011 and 31 December 2011. All included patients underwent follow-up for more than 1 year and were defined as having a successful outcome at the last post-operative visit. Success was defined as no (or only “rare”) diplopia/visual confusion for straight-ahead distances and for reading, with no greater than 10 pd. horizontal and 5.0 pd. vertical tropia in a primary position either close by or at a distance [9, 12]. Patients were excluded from the study if they had diplopia or tropia that did not meet the success criteria or if they had associated facial deformities. The study was approved by the Eye Hospital of Wenzhou Medical University Ethics Committee and adhered to the tenets of the Declaration of Helsinki.

### Clinical assessment

Full pre- and post-operative orthoptic measurements were recorded. Age, gender, education level, occupation, diagnosis, size of deviation, extraocular movement (EOM), and binocular function at the time of the survey were also recorded. Eye position was determined as tropia (constant/intermittent), phoria, or orthophoria using a cover test, and the deviation was measured using a prism and alternate cover test (PACT) at distances of 5 m and 40 cm. Combined (|dev|) was calculated with horizontal (dev_h_) and vertical (dev_v_) deviations as |dev| = √(dev_h_^2^ + dev_v_^2^) [10]. Sensory fusion was tested using the Worth 4-dot test, and stereo acuity was tested using TNO stereopsis tests. The Chinese version of the Adult Strabismus 20 (CAS-20) was also completed at the last visit to evaluate the HRQOL of the subjects [18]. The CAS-20 consists of 10 items in a psychosocial subscale and 10 items in a function subscale, each of the 20 questionnaire items was scored (0, 25, 50, 75, or 100) according to a 5-point (always, often, sometimes, rarely, or never, respectively) likert-type scale. The self-reported sense of deviation was assessed as “no deviation”, “still have some deviation”, or “still have obvious deviation” before any clinical examination was performed at the visit. Table 1 presents the characteristics assessed at the last visit.

**Table 1:** Classification of associated factors

Factor	Classification
EOM	Classified as normal, mildly abnormal (mild overaction or underaction) or obviously abnormal (obvious overaction or underaction)
Sensory fusion	Classified as normal or abnormal (suppression or diplopia)
Stereo acuity	Classified as normal stereo (≤60s of arc), partial normal stereo (120 to 480 s of arc), or none (> 480 s of arc)
Visual deficit	Defined as one or both eyes having a BCVA less than 20/60
Education level	Classified as higher education (college education and above), secondary education (community college and high school), primary education (middle school and primary school), or illiteracy
Occupation	Classified as work with people (e.g., teacher, salesperson) or work without addressing people (e.g., technician, cook, construction worker)

*BCVA best corrected visual acuity

### Analysis

For the analysis, subjects with a response of “still have some deviation” or “still have obvious deviation” were grouped as having a “self-reported sense of deviation”. The education level was classified as higher education or primary education (secondary education, primary education and illiteracy). An independent t-test was used to compare CAS scores, age, follow-up time, deviation, and the change in |dev| between the “self-reported sense of deviation” and “self-reported sense of no deviation” groups. The Chi-Square test was used to compare proportions between the groups, and if any of the groups have small numbers (ie expected cell sizes < 5), the Fisher Exact Test would be used. Moreover, multivariate analysis of variance was used to evaluate the factors associated with different characteristics. All statistical analyses were performed using the SPSS 19.0 software package (SPSS Inc., Chicago, IL, USA).

## Results

### Basic data

A total of 91 patients aged 16 to 65 years old (25.9 ± 9.7) were included in the study, of whom 41 (45%) were female. The follow-up time was 12 to 42 months (17.6 ± 4.7), and the median visual acuity was 20/20 (range: 20/100 to 20/20) in the better eye and 20/25 (range: no light perception to 20/20) in the worse eye. In addition, 30 of the 91 subjects had visual deficits in one or both eyes. Of the 91 patients, 22(24%)exhibited esotropia, 57(63%)exhibited exotropia, and 12(13%)exhibited vertical deviation prior to surgery. The horizontal deviation decreased from 44.6 ± 16.6 pd. pre-operatively to 2.3 ± 3.4 pd. post-operatively, and the vertical deviation decreased from 3.1 ± 6.8 pd. pre-operatively to 0.5 ± 1.3 pd. post-operatively, with a |dev| change of 43.4 ± 17.1 pd. after surgery. At the last visit, 53(58%)patients exhibited orthophoria, 18(20%)patients exhibited phoria, 14(15%)patients exhibited intermittent tropia, and 6(7%)patients exhibited constant tropia or dissociated vertical deviation (DVD). 7(8%)subjects had complaints of diplopia prior to surgery, and 9(10%) subjects exhibited diplopia at the periphery after surgery.

### Comparison between subjects with a sense of deviation and those without a sense of deviation

Of the 91 subjects, 69 (76%) reported no sense of deviation, and 22 (24%) reported a sense of deviation (18 patients with a sense of some deviation and 4 patients with a sense of obvious deviation). No significant difference in the patient demographics was noted between the two groups (Table 2). Furthermore, no significant differences were noted regarding pre- and post-operative deviation, changes in deviation size, and post-operative horizontal deviation between the two groups (Table 3). Subjects with a sense of deviation had larger post-operative vertical deviation and an increased prevalence of vertical deviation compared with those with no sense of deviation (Table 3).

**Table 2:** Comparison between the demographic characteristics of the two groups

	Age (y)	Follow-up time (months)	Gender		Education level		Occupation	
			Male	Female	Higher education	Primary education	Works with people	Works without addressing people
No sense of deviation (*n* = 69)	26.3 ± 10.2	17.7 ± 5.0	34	35	19	50	54	15
Sense of deviation (*n* = 22)	24.4 ± 8.1	17.5 ± 4.5	16	6	6	16	19	3
*p* value	*t* = 0.81,*p* = 0.42	t = 0.17, *p* = 0.87	*X*^*2*^ = 3.71*, p* = 0.054		*X*^*2*^ = 0.004, *p =* 0.951		*X*^*2*^ = 0.736, *p* = 0.545

**Table 3:** Comparison of strabismus characteristics between the two groups

	No sense of deviation (*n* = 69)	Sense of deviation (*n* = 22)	*P* value
Pre-operative |dev| (pd)	47.1 ± 15.4	42.7 ± 20.6	t = 1.1, *p* = 0.28
Post-operative |dev| (pd)	2.6 ± 3.5	3.1 ± 3.0	t = 0.7, *p* = 0.49
Change in |dev| (pd)	44.6 ± 16.0	39.6 ± 20.2	t = 1.2, *p* = 0.24
Post-operative horizontal deviation (pd)	2.5 ± 3.5	1.6 ± 2.9	t = 1.0, *p* = 0.30
Post-operative vertical deviation (pd)	0.06 ± 0.5	1.8 ± 2.1	t = 6.3, *p* < 0.001
CAS-20			
Psychosocial aspects	81.4 ± 14.8	70.2 ± 16.8	t = 3.0, *p* = 0.04
Functional aspects	73.2 ± 13.4	62.2 ± 16.6	t = 3.2, *p* = 0.02
Total score	77.3 ± 13.2	66.2 ± 15.0	t = 3.3, *p* = 0.001
Post-operative vertical deviation (n)			
With	1	10	*X*^*2*^ = 30.3*,* *p* < 0.001
Without	68	12	
EOM (n)			
Normal	53	14	*X*^*2*^ = 1.5*, p* = 0.22
Abnormal	16	8	
Sensory fusion (n)			
Normal	32	8	*X*^*2*^ = 0.68*, p* = 0.41
Abnormal	37	14	
Stereo function (n)			
Normal/partial Normal	34	5	*X*^*2*^ = 4.8*, p* = 0.028
None	35	17	
Visual deficit (n)			
With	22	8	*X*^*2*^ = 0.2*, p* = 0.69
No	47	14	

Table 3 indicates that subjects with no sense of deviation had better stereo function than those with a sense of deviation. No significant differences were noted in sensory fusion, EOM, or visual deficits between the two groups. Subjects with no sense of deviation had higher HRQOL scores, including scores for psychosocial aspects and functional aspects, than those with a sense of deviation.

### Factors associated with the sense of post-operative deviation

Multivariate analysis of variance was used to analyse factors including gender, education level, pre- and post-operative deviation, binocular function, and EOM. Table 4 indicates that the presence of post-operative vertical deviation was associated with a sense of deviation. Furthermore, according to the post-operative vertical deviation, 91 subjects were divided into two groups, with 81 subjects in within 2 pd. vertical group and 10 subjects in within 5 pd. vertical group, respectively. There were significant differences in sensory fusion, Stereo function, HRQOL scores between two groups. The group of within 5 pd. vertical group had worse sensory fusion, Stereo function, lower HRQOL scores.

**Table 4:** Factors associated with the sense of deviation

Source	Type III sum of squares	df	Mean square	F	*p*
Corrected model	6.623a	16	0.414	3.022	0.001
Intercept	0.275	1	0.275	2.010	0.16
Gender	0.003	1	0.003	0.019	0.89
Occupation	0.154	1	0.154	1.124	0.29
Education level	0.002	1	0.002	0.017	0.90
Direction of deviation Pre-operative	0.092	2	0.046	0.336	0.72
Pre-operative diplopia	0.039	1	0.039	0.281	0.60
Pre-operative stereo function	0.054	1	0.054	0.396	0.53
Visual deficit	0.091	1	0.091	0.667	0.42
Post-operative presence of horizontal deviation	0.011	1	0.011	0.077	0.78
Post-operative presence of vertical deviation	1.916	1	1.916	13.988	.000
EOM	0.073	1	0.073	0.530	0.47
Post-operative diplopia	0.243	1	0.243	1.773	0.19
Sensory fusion	0.018	1	0.018	0.128	0.72
Post-operative stereo function	0.306	1	0.306	2.236	0.14
Post-operative eye position	0.002	1	0.002	0.014	0.91

When success criteria of within 10 pd. horizontal and 2 pd. vertical deviations were applied in the study, 81 patients would be included in the study as with successful alignment. Of whom 37 (46%) were female, 20(25%)exhibited esotropia, 54(66%)exhibited exotropia, and 7(9%)exhibited vertical deviation prior to surgery. The horizontal deviation decreased from 47.6 ± 16.7 pd. pre-operatively to 2.4 ± 3.4 pd. post-operatively, and the vertical deviation decreased from 2.1 ± 6.0 pd. pre-operatively to 0 ± 0.2 pd. post-operatively, and only 1 subject exhibited post-operative vertical deviation. Of these 81 subjects, 68 (84%) reported no sense of deviation, and 13(16%) reported a sense of deviation. No significant differences were noted in sensory fusion, Stereo function, EOM, visual deficits, and HRQOL scores between the two groups.

## Discussion

In this study, we found that approximately 24% of adults still have a sense of deviation despite being classified as having a successful surgical outcome. Subjects with a self-reported sense of deviation exhibited worse stereo function, more vertical deviation, and worse HRQOL than those without a sense of deviation. The presence of vertical deviation was identified as a factor related to a sense of deviation.

Successful alignment criteria were not consistent in previous studies, as noted in Table 5. Here, we used criteria for a successful outcome that included motor alignment and the desired diplopia (no diplopia/visual confusion at primary and reading positions) [9]. When we used the success criteria of within 10 pd. horizontal and 2 pd. vertical deviations, the incidence of self-reported sense of deviation would be reduced from 24 to 16%. Furthermore, we didn’t find any factors associated with subjects’ sense of deviation.

**Table 5:** Comparison of motor alignment criteria between the present study and previous studies

	Motor alignment (pd)	
	Horizontal	Vertical
Our study	≤ 10	≤ 5
American Academy of Ophthalmology [1]	≤ 12	≤ 4
Keech et al. [12]	≤ 10	≤ 5
Zhang et al. [15]	≤ 10	≤ 2
Carruthers et al. [19]	≤ 10	≤ 10
Hertle et al. [13]	≤ 12	≤ 5
Beauchamp et al. [14]	≤ 8	≤ 2
Hatt et al. [16]	≤ 10	≤ 10

Our study confirmed that surgical outcome assessment reveals different perspectives between patients and physicians. Beauchamp et al. found that a difference in severity ratings between patients and physicians improved after surgery [17]. A lower percentage of subjects reported a sense of deviation after surgery in our study than in a report by Satterfield et al., where in 84% of subjects reported a sense of deviation. However, this group did not assess the actual deviation of the subjects, and the subjects reported an inability to perform stereo tasks and an inability to use both eyes together in that study [5]. Moreover, 23% (5/22) and 36% (8/22) of subjects with a sense of deviation had normal/partial normal stereo function and sensory fusion, respectively, in our study.

We report that changes in deviation and post-operative horizontal deviation did not differ between subjects in the two groups. A previous study demonstrated no significant correlation between psychosocial distress and deviation changes after surgery, whereas social anxiety and social avoidance (assessed using the Derriford Appearance Scale (DAS-24)) were both correlated with post-operative deviation and subjective strabismus severity (assessed via visual analogue scale (VAS)). However, the correlation between objective deviation size and subjective strabismus severity was not assessed in that study [7].

Female gender and a lower socioeconomic status have been associated with worse psychosocial aspects pre-operatively and/or post-operatively [10, 20]. We identified no significant difference in demographics, e.g., gender, age, occupation and education level, between subjects with and without a sense of deviation, although socioeconomic status as well as social anxiety was not reviewed in the current study. Social anxiety levels have also been reported be related to post-operative HRQOL in adult patients [21].

Post-operative HRQOL, as assessed using the CAS-20, was enhanced in subjects with no sense of deviation compared with those with a sense of deviation in the present study. This finding implies that post-operative HRQOL assessment can be applied as a criterion to evaluate the subjective outcome of surgery. As recent studies have suggested that motor alignment criteria cannot comprehensively represent a patient’s post-operative status, this information combined with HRQOL tests may serve as a more useful method to evaluate successful outcome judgements [9, 22].

The presence of vertical deviation was identified as a factor related to the sense of deviation in the current study. Compared with the post-op within 2 pd. vertical group, the post-op within 5 pd. vertical group had worse sensory fusion, worse stereo function and lower HRQOL scores. In clinical work, these 3 to 5 pd. vertical deviations (including a certain degree of phoria) were not obvious in appearance when combined with multiplanar deviations, especially with a large angle horizontal deviation. Although approximately 45% (10/22) of subjects with a self-reported sense of deviation exhibited a small vertical deviation, it is possible that this small vertical deviation could lead to asthenopia [23] or abnormal head posture [24]. However, asthenopia and abnormal head posture were not assessed in the current study. This finding also implied that even a small vertical deviation should be treated comprehensively.

There were some limitations to this study. First, we used criteria for a successful outcome that included motor alignment (no greater than 10 pd. horizontal and 5 pd. vertical deviations) and the desired diplopia (no diplopia/visual confusion at primary and reading positions), whereas some previous studies use a lower threshold for vertical alignment (2 pd). When we used the stricter definition (within 10 pd. horizontal and 5 pd. vertical deviations), we didn’t find any factors associated with subjects’ sense of deviation. Further evaluations should be combined with more factors in future studies.

Second, although social anxiety levels have been reported to be related to the judgement of post-operative HRQOL in adult patients [21] and although psychosocial characteristics are thought to play a more important role than clinical aspects in the well-being of populations with strabismus, [25] we did not review psychosocial features, e.g., social anxiety and depression, we also did not review other factors that may influence the patient’s perspective, such as palpebral fissure width, persistent redness of the conjunctiva in the current study.

## Conclusions

In sum, approximately one-fourth of adults with successful surgical outcomes experience a sense of deviation. Subjects with a self-reported sense of deviation had worse stereo function, a larger vertical deviation size and lower HRQOL scores. The presence of 3 to 5 pd. vertical deviation would be the main factor related to the sense of deviation after successful surgery. We should use the stricter definition (within 10 pd. horizontal and 5 pd. vertical deviations) as the success criteria to assess the surgical outcome in clinic work.

## Data Availability

The datasets used and analysed during the current study are available from the corresponding author on reasonable request.

## Abbreviations

CAS-20: The Chinese version of the Adult Strabismus 20
EOM: Extraocular movement
HRQOL: Health-related quality of life
PACT: A prism and alternate cover test
